# Antiviral Activity of *Ecklonia cava* Extracts and Dieckol Against Zika Virus

**DOI:** 10.3390/ijms252413694

**Published:** 2024-12-21

**Authors:** Eun-A Kim, Nalae Kang, Jun-Ho Heo, Areumi Park, Seong-Yeong Heo, Hyun-Soo Kim, Soo-Jin Heo

**Affiliations:** 1 Jeju Bio Research Center, Korea Institute of Ocean Science and Technology (KIOST), Jeju 63349, Republic of Korea; euna0718@kiost.ac.kr (E.-A.K.); nalae1207@kiost.ac.kr (N.K.); unknown0713@kiost.ac.kr (J.-H.H.); areumi1001@kiost.ac.kr (A.P.); syheo@kiost.ac.kr (S.-Y.H.); 2 Department of Seafood Science and Technology, The Institute of Marine Industry, Gyeongsang National University, Tongyeong 53064, Republic of Korea; gustn783@gnu.ac.kr; 3 Department of Marine Biology, University of Science and Technology, Daejeon 34113, Republic of Korea

**Keywords:** *Ecklonia cava*, dieckol, Zika virus, Vero E6 cells, in silico assays

## Abstract

*Ecklonia cava* and its major compound dieckol, both natural marine products, possess antioxidant, anti-inflammatory, and metabolic-regulating effects. Zika virus (ZIKV), an arbovirus from the *Flaviviridae* family, is transmitted by mosquitoes and causes serious illnesses in humans. This study aimed to evaluate the anti-ZIKV potential of *Ecklonia cava* and dieckol. The antiviral activity of *Ecklonia cava* extract (ECE), prepared with 80% ethanol, was assessed in ZIKV-infected Vero E6 cells through MTT assay, plaque assay, and quantitative polymerase chain reaction (qPCR), demonstrating no cytotoxicity and a significant reduction in viral titers and ZIKV mRNA levels. In addition, ECE decreased the expression of tumor necrosis factor-α and interferon-induced protein with tetratricopeptide repeats in the ZIKV-infected cells. Dieckol, the primary active compound in ECE, exhibited potent anti-ZIKV activity, with a half maximal inhibitory concentration (IC_50_), value of 4.8 µM. In silico molecular docking analysis revealed that dieckol forms stable complexes with key ZIKV proteins, including the envelope, NS2B/NS3, and RNA-dependent RNA polymerase (RdRp) protein, exhibiting high binding energies of −438.09 kcal/mol, −1040.51 kcal/mol, and −1043.40 kcal/mol, respectively. Overall, our findings suggest that ECE and dieckol are promising candidates for the development of anti-ZIKV agents.

## 1. Introduction

The Zika virus (ZIKV), a member of the genus *Flavivirus*, is transmitted through mosquito bites and causes severe diseases in humans and animals [1,2]. Viruses from the *Flaviviridae* family, such as Zika, Dengue, Yellow fever, and West Nile viruses, originate in tropical and subtropical regions and have spread globally, posing a major public health concern [1,2,3]. In particular, climate change is anticipated to increase overall temperature, thereby increasing the mosquito population, with potential consequences for humans [4] Among these, ZIKV is strongly associated with Guillain–Barré syndrome, an autoimmune disorder in which the immune system attacks the nervous system, leading to nerve and muscle inflammation [3]. Furthermore, ZIKV infection in pregnant women can result in brain calcifications, microcephaly, and other congenital malformations in fetuses and newborns [1,5,6]. Despite continuous efforts to develop antiviral drugs for the treatment of ZIKV, a critical global shortage of effective antiviral agents persists [3,6]. For this reason, the World Health Organization has classified the diseases with epidemic potential and/or a lack of or insufficient countermeasures and identified the ZIKV as a pathogen that is prioritized for R&D in public health emergency contexts [7]. Between 1981 and 2019, a total of 185 antiviral drugs were approved, of which 28.65% were derived from natural products or their derivatives [2,8]. Therefore, utilizing compounds derived from natural products represents a promising approach for the development of anti-ZIKV therapeutics.

ZIKV consists of three structural proteins, namely capsid, pre-membrane, and envelope proteins, and seven nonstructural proteins: NS1, NS2A, NS2B, NS3, NS4A, NS4B, and NS5 [9,10]. The envelope protein is a crucial membrane protein essential for viral structural assembly, attachment, and penetration into the host cell [10]. Previous studies have demonstrated the effectiveness of anti-ZIKV drugs targeting NS2B/NS3 and NS5 proteins [10,11]. NS2B/NS3, a trypsin-like serine protease, is considered a promising target for anti-flavivirus drug design due to its critical role in proteolysis and viral replication [9,12]. NS5, the largest enzyme of ZIKV, consists of two distinct domains: the RNA-dependent RNA polymerase (RdRp) domain at the C-terminus, and the methyltransferase domain at the N-terminus [10,11]. The NS5 RdRp domain plays a pivotal role in viral RNA synthesis and replication [10,11].

The innate immune system serves as the initial line of defense against viral infections and is essential for mounting an appropriate immune response [13]. Cytokines such as tumor necrosis factor a (TNF-α), interleukin (IL)-1, IL-6, and IL-18 are known as immune effectors that stimulate antiviral responses in targeted cells [13]. Among the cytokines, TNF-α has the potential to cause significant damage to healthy host tissues due to its critical role in regulating cell death pathways [14,15]. Furthermore, interferon-induced proteins with tetratricopeptide repeats (IFITs), which are regulated by interferon signaling, play multiple roles in blocking viral translation and replication, making them prominent markers of virus-infected cells [3,16].

Marine seaweed is a natural product known to have potential antiviral properties [17]. *Ecklonia cava*, a major edible brown seaweed, possesses a range of beneficial biological activities, including antioxidant, anti-inflammatory, anticancer, antihypertensive, antineuroprotective, antimicrobial, and antiviral properties [18,19,20]. Among its unique compositions (polyphenols, polysaccharides, peptides, carotenoids, and steroids), polyphenols have demonstrated effectiveness against various types of viruses, including coronaviruses, influenza, rotavirus, ZIKV, and Dengue virus [1,2,18,20,21,22]. *Ecklonia cava* extracts and/or *E. cava*-derived polyphenols have demonstrated antiviral properties against the hepatitis A virus, porcine epidemic diarrhea virus, hemorrhagic septicemia virus, and human immunodeficiency virus type-1 [21,23,24,25]. Furthermore, dieckol, a major constituent of *E. cava* polyphenols, possesses antiviral activity. However, there are no studies on the anti-ZIKV activity of *E. cava* and dieckol. Therefore, we aimed to evaluate the anti-ZIKV activity of *E. cava* ethanol extract (ECE) and dieckol on ZIKV-infected Vero E6 cells.

## 2. Results

### 2.1. Antiviral Effects of E. cava Extract in ZIKV-Infected Vero E6 Cells

**Figure 1:**
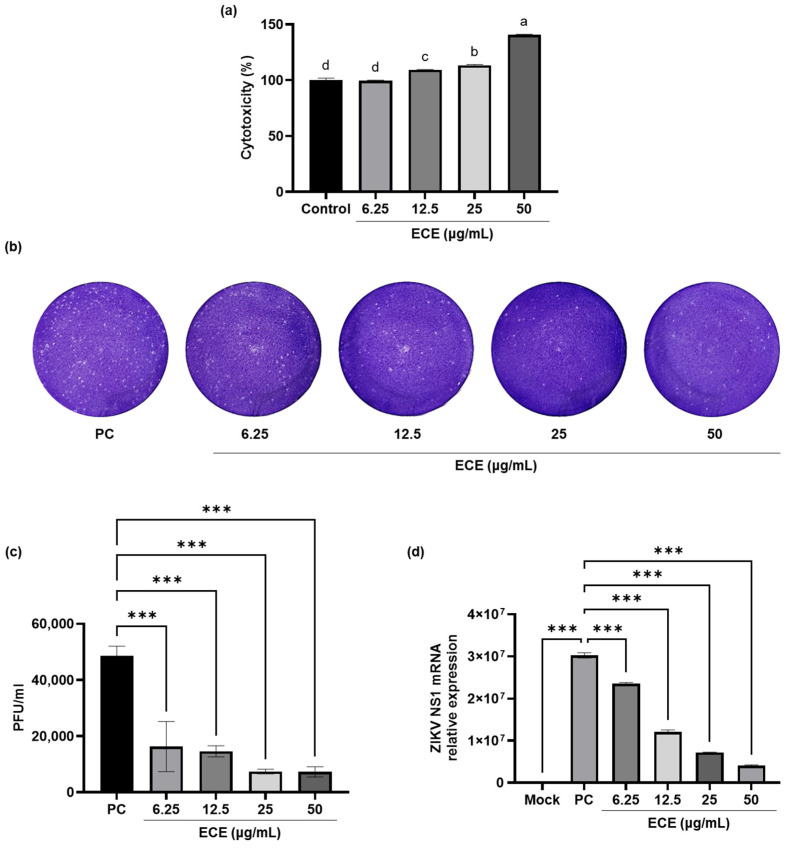
Anti-Zika virus activity of *Ecklonia cava* ethanol extract in virus-infected Vero E6 cells: (**a**) the cytotoxicity of ECE in Vero E6 cells using MTT assay; (**b**) plaque image; (**c**) virus was measured using plaque assay; (**d**) cell lysates were extracted, and the mRNA level of Zika virus NS1 was analyzed using qPCR. Values are expressed as mean ± SD of triplicate experiments. Different letters (a, b, c, d) indicate statistical significance (*p* < 0.05) and *** *p* < 0.001 indicate significant differences from PC. ECE: *Ecklonia cava* ethanol extract, qPCR: quantitative polymerase chain reaction, SD: standard deviation, MTT: (3-(4,5-dimethylthiazol-2-yl)-2,5-diphenyltetrazolium Bromide), PC: virus groups.

First, the cytotoxicity of ECE was evaluated at concentrations of 6.25 to 50 μg/mL in Vero E6 cells through an MTT assay (Figure 1a), and the cell viabilities of Vero E6 cells after treatment with ECE at concentrations of 6.25, 12.5, 25, and 50 μg/mL were 99.60%, 109.19%, 113.16%, and 140.54%, respectively, compared to the control groups (0.05% DMSO solution-treated groups). The increasing concentrations of ECE demonstrated a high level of cell viability. Thus, a 6.25 to 50 μg/mL concentration range was selected for the subsequent experiments. The anti-ZIKV effect was determined using a plaque assay (Figure 1b,c). Infection with 0.01 multiplicity of infection (MOI) in virus groups (PC; ZIKV-only infection groups) resulted in a plaque-forming virus titer of 48,500 PFU/Ml; however, treatment with ECE reduced the virus titer to 16,300, 14,600, 7400, and 7300 PFU/mL at concentrations ranging from 6.25 to 50 μg/mL, respectively. Additionally, following infection, ZIKV viral RNA was detectable in the PC group differently from the mock group (0.05% DMSO solution-treated groups) (Figure 1d). Notably, ECE treatment markedly decreased ZIKV mRNA expression levels.

### 2.2. Effect of E. cava Extract on Tumor Necrosis Factor (TNF)-α and IFIT mRNA Levels in ZIKV-Infected Vero E6 Cells

**Figure 2:**
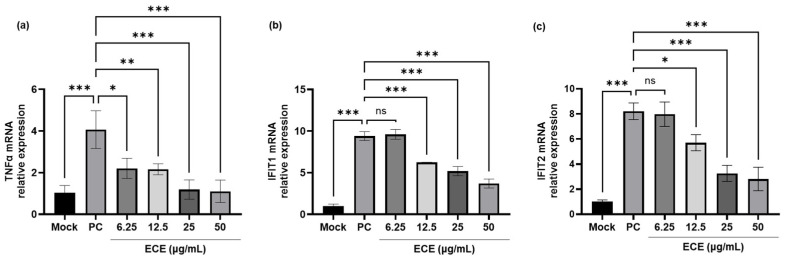
Effects of *Ecklonia cava* ethanol extracts on TNF-α and IFIT 1 and IFIT2 mRNA levels in Zika virus-infected Vero E6 cells. Cell lysates were extracted, and the mRNA levels of (**a**) TNF-α, (**b**), and (**c**) IFIT2 were analyzed using qPCR. Values are expressed as mean ± SD of triplicate experiments. ns: not significant, * *p* < 0.05, ** *p* < 0.005, *** *p* < 0.001 indicate significant differences from PC. TNF-α: tumor necrosis factor-α, IFIT: interferon-induced protein with tetratricopeptide repeats, qPCR: quantitative polymerase chain reaction, SD: standard deviation.

The ECE showed anti-ZIKV activity in the plaque assays. Therefore, we assessed the mRNA levels of TNF-α and IFITs using quantitative polymerase chain reaction (qPCR). As shown in Figure 2a, viral inoculation led to an increase in TNF-α mRNA expression, a molecule that can cause significant cellular damage. However, treatment with ECE simultaneously with viral inoculation reduced the TNF-α mRNA expression, suggesting that ECE modulates the immune response and inhibits TNA-α expression. In contrast, as shown in Figure 2b,c, viral inoculation increased the mRNA expression levels of IFITs, which play a role in blocking viral translation and replication. This increase was significantly reduced by ECE treatment, indicating that ECE may directly destroy the virus or inhibit viral translation, thereby reducing the viral particle load. Overall, these results suggest that ECE exerts antiviral activity in ZIKV-infected Vero E6 cells by modulating TNF-α and IFIT-mediated immune responses and decreasing viral particle levels.

### 2.3. Antiviral Effects of Dieckol in ZIKV-Infected Vero E6 Cells

**Figure 3:**
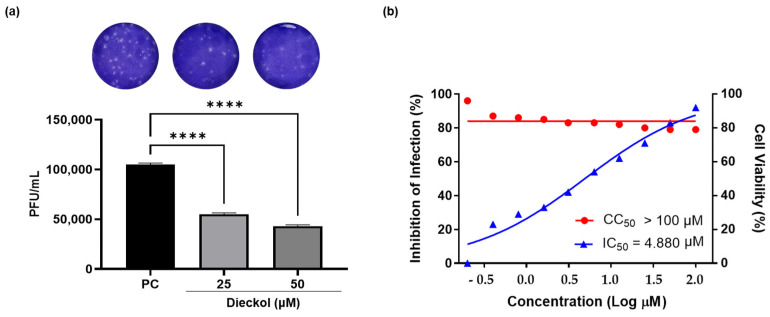
Anti-Zika virus activity of dieckol in virus-infected Vero E6 cells: (**a**) plaque image and titer were measured using plaque assay at 25 and 50 µM; (**b**) cytotoxicity and anti-Zika virus profiles (half-maximal inhibitory concentration; IC_50_) of dieckol in Vero E6 cells at various concentrations 0.20–100 µM using MTT and plaque assay, respectively. Values are expressed as mean ± SD of triplicate experiments. **** *p* < 0.0001 indicates significant differences from PC. MTT: MTT: (3-(4,5-dimethylthiazol-2-yl)-2,5-diphenyltetrazolium bromide), SD: standard deviation, PC: virus groups.

We further identified the ECE components that exhibited antiviral activity. Dieckol is the main component of *E. cava* and has shown antiviral effects against murine norovirus and SARS-CoV-2 [26,27,28,29]. Consequently, the main compound of ECE was verified through HPLC analysis, which revealed that dieckol is the predominating compound (Figure S1). Therefore, the anti-ZIKV activity of dieckol was evaluated (Figure 3). First, the inhibition of plaque formation was confirmed at 25 and 50 μM of dieckol. As shown in Figure 3a, dieckol inhibited ZIKV titers to 62,000 and 50,000 PFU/mL at 25 and 50 μM, respectively, compared to that of the PC group. Further, we conducted ZIKV inhibition experiments with various concentrations of 0.20–100 μM (Figure 3b). The cytotoxic concentration (CC_50_) of dieckol was assessed using an MTT assay. Dieckol showed approximately 79% cell viability at 50 and 100 μM, while concentrations below 25 μM showed cell viability of more than 80% in Vero E6 cells (Figure 3a). Therefore, it was determined that the CC_50_ values were greater than 100 μM. As shown in Figure 3b, dieckol inhibited ZIKV infection in a dose-dependent manner by reducing plaque formation. The inhibitory activity of dieckol against ZIKV increased in a concentration-dependent manner, with an IC_50_ value of 4.88 μM.

### 2.4. Virucidal Effect of Dieckol Against ZIKV in Vero E6 Cells

**Figure 4:**
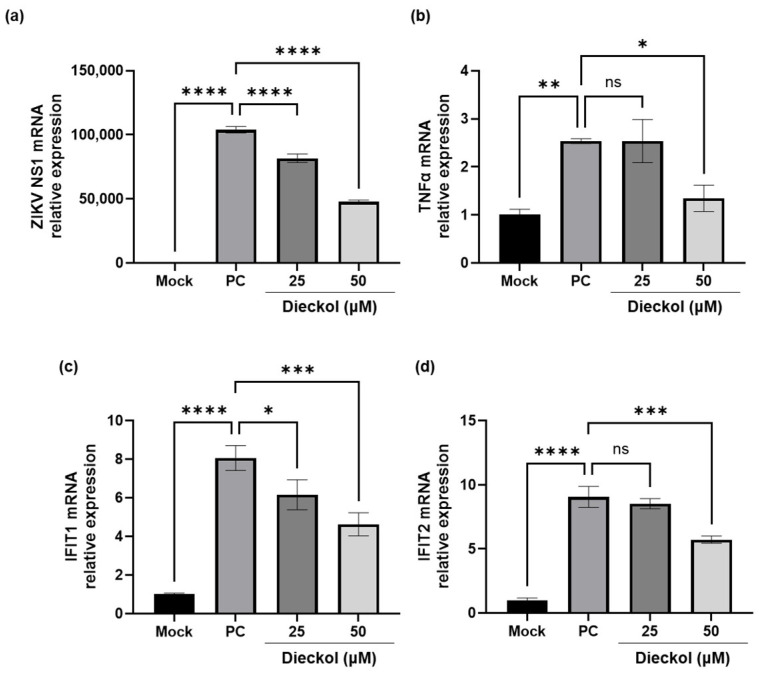
The virucidal effect of dieckol: Cell lysates were extracted, and the mRNA levels of (**a**) ZIKV NS1, (**b**) TNF-α, (**c**), IFIT1, and (**d**) IFIT2 were analyzed using qPCR. Values are expressed as mean ± SD of triplicate experiments. ns: not significant, * *p* < 0.05, ** *p* < 0.01, *** *p* < 0.001, **** *p* < 0.0001 indicate significant differences from PC. PC: virus groups, TNF-α: tumor necrosis factor-α, IFIT: interferon-induced protein with tetratricopeptide repeats, qPCR: quantitative polymerase chain reaction, SD: standard deviation.

Antiviral mechanisms include the inhibition of viral infection, such as blocking viral entry and/or replication, as well as the regulation of the innate immune system. In contrast, the virucidal effect involves a direct interaction with and the physical disruption of viral particles. Viral inactivation can influence antiviral activity by reducing the number of viruses entering the cells, thereby lowering viral quantification. To confirm the direct interaction and physical disruption of ZIKV particles, the potential virucidal effect of dieckol was further validated by evaluating its impact on the mRNA expression levels of ZIKV NS1, TNF-α, and IFITs using qPCR (Figure 4). As shown in Figure 4a, the PC groups exhibited a marked increase in ZIKV mRNA levels differently from the mock group. In contrast, treatment with dieckol at concentrations of 25 and 50 μM markedly reduced ZIKV NS1 mRNA levels. Dieckol treatment at a high concentration (50 μM) significantly reduced the mRNA level of TNF-α compared to the PC groups, as shown in Figure 4b. The mRNA levels of antiviral proteins IFIT1 and IFIT2 were markedly elevated in the PC groups compared to the mock groups. Dieckol demonstrated a concentration-dependent decrease in the mRNA expression levels of IFIT1 and IFIT2, with particularly notable suppression observed at a concentration of 50 μM (Figure 4c,d). The results demonstrated that dieckol possesses antiviral activity by directly disrupting the virus, which in turn reduces the immune response. We have reorganized the paragraph to improve clarity and coherence.

### 2.5. Molecular Docking Analysis of the Interaction of Dieckol with Three Major ZIKV Proteins

**Figure 5:**
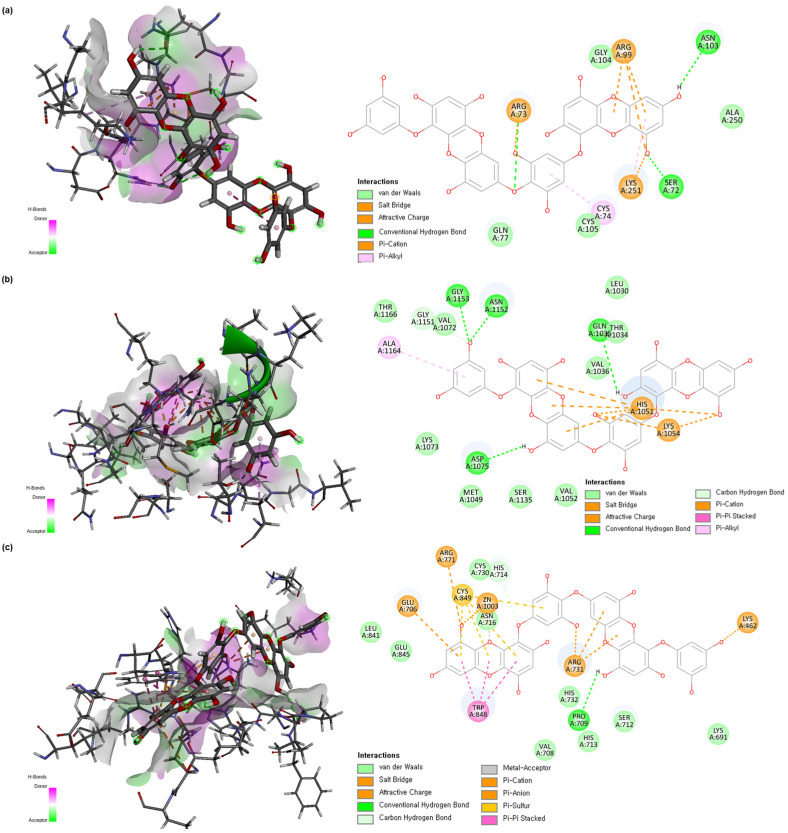
In silico docking assay for the docking poses of dieckol to the three main Zika virus proteins: (**a**) the envelope protein, (**b**) NS2B/NS3, and (**c**) RdRp.

Docking analysis was performed to characterize the interaction energy between dieckol and the major ZIKV proteins. Therefore, we identified three proteins as key molecular targets of ZIKV, namely the envelope protein, NS2B/NS3, and RdRp, using the Chemistry at Harvard Macromolecular Mechanics (CHARMM)-based DOCKER (CDOCKER) energy, as well as interaction energy, and calculated binding energy (Figure 5). Previous studies have shown the binding site for the envelope protein in the fusion loop, the binding site for NS2B/NS3 at the site that interacts with adenosine triphosphate, and the binding site for RdRp in the priming loop [30,31,32]. Molecular docking was performed by defining binding sites based on previous studies. The 3D and 2D interaction diagrams of the dieckol and envelope protein complex showed a network of various interactions between the following residues: SER72 (conventional hydrogen bond), ARG73 (conventional hydrogen bond, salt bridge, and attractive charge), CYS74 (pi–alkyl with one ring), ARG99 (pi–cation with two rings, and attractive charge), ASN103 (conventional hydrogen bond), and LYS251 (salt bridge, attractive charge, and pi–alkyl with one ring). Therefore, the dieckol and envelope protein indicated 35.4185, 75.4367, and −438.087 kcal/mol -CDOCKER energy, -CDOCKER interaction energy, and binding energy, respectively (Figure 4a, Table 1). Moreover, dieckol demonstrated interactions with amino acids of NS2B/NS3, including GLN1035 (conventional hydrogen bond), HIS1051 (pi–cation with three rings, attractive charge, and salt bridge), LYS1054 (salt bridge and attractive charge), ASP1075 (conventional hydrogen bond), GLY1151 (carbon–hydrogen bond), ASN1152 (conventional hydrogen bond), GLY1153 (conventional hydrogen bond), and ALA1164 (pi–alkyl with one ring) and revealed the -CDOCKER energy, -CDOCKER interaction energy, and binding energy, which were found to be 52.9245 kcal/mol, 101.435 kcal/mol, and −1040.51 kcal/mol, respectively (Figure 4b, Table 1). Furthermore, the dieckol and RdRp complex exhibited interactions with various amino acids, including LYS462 (salt bridge and attractive charge), GLU706 (pi–anion with one ring), PRO709 (conventional hydrogen bond), HIS 714 (carbon–hydrogen bond), ARG731 (pi–cation with two rings, salt bridge, and attractive charge), ARG771 (attractive charge), TRP848 (pi–pi-stacked with three rings), CYS849 (pi–sulfur with four rings), ZN1003 (attractive charge and metal acceptor). Moreover, -CDOCKER energies indicated a value of −22.7957 kcal/mol, -CDOCKER interaction energy indicated a value of 41.2826 kcal/mol, and binding energy indicated a value of −1043.4 kcal/mol (Figure 4c, Table 1). These results indicated that dieckol plays an important role in the interaction with Zika proteins, suggesting that all the functional groups of dieckol, including phenol rings, oxygens, and hydrogens, affected the interaction with the three proteins.

## 3. Discussion

The Zika virus, a flavivirus capable of infecting both humans and animals, currently lacks effective antiviral therapies. Thus far, no drugs have been approved for the treatment of ZIKV infection. Therefore, exploring a range of potential drugs is essential. The present study was performed to identify potential pharmaceutical agents derived from marine algae that could be used for ZIKV infection treatment.

In this study, we evaluated the anti-Zika virus activity of the marine natural product *Ecklonia cava* and its major bioactive compound, dieckol.

The cytotoxicity of ECE was evaluated in Vero E6 cells, showing high cell viability (99.60–140.54%) at concentrations of 6.25–50 μg/mL, which were selected for further experiments. ECE exhibited a dose-dependent anti-ZIKV effect, with viral titers in the ZIKV-only infection group (PC) measured at 48,500 PFU/mL, which were significantly reduced to 16,300 PFU/mL following treatment with ECE at a concentration of 6.25 μg/mL. Additionally, ECE significantly decreased ZIKV mRNA expression levels. Furthermore, extracts of *Canistrocarpus cervicornis* and *Dictyota menstrualis*, brown seaweeds from the Brazilian coast, inhibited ZIKV by 42% with half-maximal inhibitory concentration (IC_50_) values of 2.2 µg/mL and 20 µg/mL, respectively [33,34]. Also, the IC_50_ values indicated for *Aploia theiformis* (green tree) and *Psiloxylon mauritianum* (a species of flowering plant) were 100 µg/mL and 19.5 µg/mL, respectively [35]. In this study, ECE inhibited 66.4% of ZIKV at 6.25 µg/mL, indicating considerable antiviral activity comparable to that of the aforementioned brown seaweed extracts and plants.

The innate immune system serves as the first line of defense against viral infections, with cytokines like TNF-α and IFITs playing critical roles in antiviral responses [13]. ECE reduced the mRNA levels of TNF-α, IFIT1, and IFIT2 in a concentration-dependent manner, as confirmed by qPCR. These findings suggest that ECE inhibits the apoptotic process and blocks viral translation and replication, contributing to its TNF-α and IFIT-mediated antiviral effects in ZIKV-infected Vero E6 cells.

Therefore, we confirmed the anti-ZIKV effects of ECE and further evaluated the anti-ZIKV activity of its main compound. *Ecklonia cava* contains many active substances, including polyphenols, polysaccharides, fatty acids, and peptides, among which polyphenols account for the largest proportion of organic solvent extracts [18,19,20]. The major polyphenols present in ethanol and methanol extracts of *E. cava* are phloroglucinol, eckol, and dieckol [19,36]. Furthermore, the components of *E. cava* are known to include 6,6-bieckol, phloroeckol, phlorofucofuroeckol A, dibenzodioxin-fucodiphloroethol, and 8,8′-bieckol [19,20]. Additionally, ECE showed the main compound of dieckol by HPLC analysis. Dieckol inhibited concentration-dependent inhibitory activity against ZIKV, with an IC_50_ value of 4.88 µM, while maintaining a CC_50_ value exceeding 100 µM, thereby demonstrating selective and potent antiviral activity. Furthermore, it also showed virucidal effects. Epigallocatechin gallate, the most abundant polyphenol in green tea, and delphinidin, a plant pigment, showed complete inhibition of ZIKV infection at approximately 60% and 40% at a concentration of 10 μM, respectively [2]. Baicalin isolated from *Scutellaria balcalensis* and *Scutellaria lateriflora* and berberine isolated from *Berberis valgaris* demonstrated anti-ZIKV activity with 50% effective concentration of viral replication (EC_50_) values 14 μM to 39.06 μM, respectively [37]. Crenatoside isolated from *Tecoma stans var* exhibited an EC_50_ value of 37.78 μM [5]. The antiviral drug ribavirin showed an EC_50_ value of 386.85 μM, while the synthetic antiviral arbidol demonstrated an IC_50_ value of 15 μM following a 1 h pretreatment [5,38]. Sofosbuvir, another antiviral agent, displayed anti-ZIKV activity with an IC_50_ value of 4 μM [39]. Furthermore, fucoxanthin, a marine carotenoid, inhibited ZIKV replication by approximately 84% at a concentration of 12.5 μM [3]. In comparison with previously reported natural product-derived compounds and approved antiviral drugs, dieckol demonstrates excellent anti-ZIKV activity, underscoring its potential as a highly effective antiviral agent against ZIKV. In silico studies are being utilized for the exploration of potential candidates and experimental validation. Accordingly, the interaction energy between dieckol and the major ZIKV proteins such as the envelop protein, NS2B/NS3, and RdRp, was analyzed. Dieckol demonstrated a critical role in its interaction with ZIKV proteins, with its functional groups, including phenol rings, oxygen atoms, and hydrogen atoms, significantly contributing to its binding affinity with the three target proteins. Furthermore, the interaction energy of dieckol with the three major ZIKV proteins was superior to that of fucoxanthin, previously isolated from brown algae [3]. The binding energy of fucoxanthin and ZIKV envelope protein or RdRp complex was determined to be −151.45 and −290.92 kcal/mol, respectively [3]. Molecular docking analysis revealed that dieckol exhibited stronger binding energy for the envelope, NS2B/NS3, and RdRp proteins. Further studies can be conducted to investigate the mechanisms and resistance mechanisms of anti-ZIKV activity of dieckol in an in vitro model, as well as the efficacy of ECE and dieckol in vivo by conducting animal model studies. Furthermore, additional anti-ZIKV studies will be conducted considering the potential compounds from ECE.

## 4. Materials and Methods

### 4.1. Isolation of Dieckol from E. cava

*Ecklonia cava*, a brown alga, was collected from the coast of Jeju Island in South Korea. It was washed three times with tap water to remove salts, parasites, and sand attached to the surface and stored in a refrigerator at −20 °C. The frozen samples were freeze-dried and homogenized using a grinder for extraction. The ground samples were further extracted with 80% ethanol (ECE). Dieckol was isolated and purified according to a method reported by Heo et al. (2009) [40]. For cell-based analysis, the dried ECE and dieckol were dissolved in dimethyl sulfoxide (DMSO) and subsequently used in experiments, with the final concentration of DMSO in the culture medium adjusted to 0.05%. For HPLC analysis, ECE was dissolved in methanol and filtered through a 0.22 μm PVDF membrane filter. ECE was subjected to analysis using an Alliance 2695 system and a 2998 photodiode array detector (Waters Corporation, Milford, MA, USA), with a YMC-Triart C18 column (4.6 × 250 mm, 5 μm, YMC Co., Ltd., Kyoto, Japan). The column was eluted in gradient mode with a mobile phase solvent system comprising water with 0.1% formic acid (A) and acetonitrile with 0.1% formic acid (B). The analysis conditions were as follows: 0–10 min: 95:5, *v*/*v*; 10–60 min: 95:5 → 0:100, *v*/*v*; 60–70 min: 0:100, *v*/*v* with a flow rate of 1 mL/min. The absorbance was monitored at 230 nm.

### 4.2. Cell Culture and Cytotoxicity

Vero E6 cells were purchased from the American Type Culture Collection (ATCC CRL-1596) and cultured in Dulbecco’s modified Eagle medium (DMEM) supplemented with 10% fetal bovine serum (FBS) and 1% penicillin–streptomycin and maintained at 37 °C in a 5% CO_2_ incubator. ZIKV was supplied by the National Culture Collection for Pathogens from the Korea Centers for Disease Control and Prevention (NCCP NO. 43280, isolating a virus from a patient with ZIKV infection by infecting mammalian cells). The cytotoxicity of ECE and dieckol was assessed using the MTT assay [41]. Briefly, Vero E6 cells were seeded at a concentration of 1.4 × 10^4^ cells per 96-well plate until the cells formed a monolayer (100% confluence). ECE (6.25–50 μg/mL) and dieckol (0.20–100 μM) samples were tested at various concentrations, with cells incubated for 24 h. Afterward, the MTT solution was added and incubated for an additional 3 h. The supernatant was then removed, and the formazan crystals were dissolved in DMSO. The optical density was then measured at 570 nm using a microplate reader.

### 4.3. Plaque Assay

The antiviral effects of the ECE and dieckol samples were evaluated using a plaque assay. A monolayer of Vero E6 cells in a 6-well plate was washed before adding DMEM supplemented with 2% FBS. The cells were treated by simultaneously inoculating them with various concentrations of samples (ECE 6.25–50 μg/mL or dieckol 0.20–100 μM) and ZIKV (0.01 MOI) for 48 h. The supernatant was serially diluted by a factor of 10 in a 1.5 mL tube containing only DMEM. Further, the diluted samples were treated in the 6-well plates with 1 × 10^6^ Vero E6 cells per well and incubated in DMEM supplemented with 10% FBS until the cells reached 100% confluence, forming a monolayer. The cells were incubated at 37 °C in a 5% CO_2_ incubator for 90 min with gentle shaking every 15 min to facilitate virus adsorption. Following the adsorption process, the inoculum was removed from the cells, and 3 mL of a pre-mixed DMEM-F12-2% oxoid agarose solution (7:3) was added. The cells were then incubated at 37 °C in a 5% CO_2_ incubator for 5 days. Further, the cells were fixed with 1 mL of 4% formaldehyde for 1 h, and the agarose gel was removed. Subsequently, 1 mL of 0.1% crystal violet was added, and the mixture was incubated for 30 min at room temperature. The crystal violet solution was discarded, and the cells were washed with PBS and allowed to dry. Viral titers were calculated by counting the number of plaques according to the following formula [42]:

PFU/mL = Number of plaques/(dilution factor × volume of diluted virus/well).

### 4.4. qPCR

qPCR analysis was performed as described by Kang et al. [3]. Vero E6 cells treated with the test samples were prepared as described in Section 4.3. Total RNA was extracted using TRIzol reagent following the manufacturer’s instructions. Complementary DNA was synthesized from 2 µg of RNA using the Prime Script RT Reagent Kit. The reaction mixture, containing 1x RT buffer mix and 20x RT enzyme mix, was incubated at 37 °C for 60 min, followed by enzyme inactivation at 95 °C for 5 min. qPCR analysis was performed using the SYBR green-based detection method with a Quant Studio 3 qPCR system (Applied Biosystems, Thermo, Waltham, MA, USA) with a modified method and reagents. All primers used are listed in Table 2.

### 4.5. Virucidal Assay

Dieckol was diluted 2X in DMEM, and the virus stock was prepared at a concentration of 1 × 10^5^ PFU/mL in DEME. Equal volumes of the diluted dieckol and virus stock were mixed in a 1:1 ratio and incubated at 37 °C in a 5% CO_2_ incubator for 1h. Following incubation, the Vero E6 cells were treated by simultaneously inoculating them with samples for 48 h. Subsequently, the cells were collected, and qPCR was performed as described in detail in Section 4.4.

### 4.6. Statistical Analysis

All data were generated in triplicate and expressed as means ± standard deviation (SD). One-way analysis of variance (ANOVA) was used to compare the datasets followed by Tukey’s multiple comparison test using GraphPad Prism software version 9 (GraphPad Software, San Diego, CA, USA). Statistical significance was set at *p* < 0.05.

### 4.7. The 3D Structure of Dieckol

The structural data file of dieckol was obtained from PubChem (CID: 3008868), and geometry optimization of the 3D structure was confirmed using the ligand preparation, energy minimization, and conformation generation protocols of Discover Studio 2024.

### 4.8. Molecular Docking Analysis of the Protein and Dieckol

The objective of the molecular docking analysis was to assess the structural effects of dieckol on the three main proteins using the CDOCKER protocol based on CHARMM and to calculate the binding energy tolls in Discovery Studio 2023. The 3D structures of the main ZIKV envelope protein (PDB ID: 5JHM), NS2B/NS3 protein (PDB ID: 5LC0), and RdRp protein (PBD ID: 5TFR) were obtained from the Protein Data Bank (PDB). The docking poses of dieckol on the three main proteins are illustrated in the 2D and 3D crystal structures. The binding sites of the three proteins were determined based on the findings of a previous study [3].

## 5. Conclusions

In conclusion, ECE effectively inhibited viral titers and ZIKV mRNA levels in ZIKV-infected Vero E6 cells. Additionally, it demonstrated antiviral activity by suppressing the production of TNF-α and IFIT. Notably, dieckol, a major bioactive compound in ECE, exhibited significant anti-ZIKV effects and virucidal effects. The in silico molecular docking results predicted that dieckol would exhibit high binding energy to the envelope, NS2B/NS3, and RdRp proteins. In particular, the fusion loop (residues 98–109) of the envelope protein is responsible for the membrane fusion of host cells and viral membranes, which occurs during the process of virus entry. The loop is highly conserved in flaviviruses, including ZIKV. Following the in silico analysis results, dieckol was observed to bind to the fusion loop, thereby inhibiting ZIKV attachment and entry into host cells. Therefore, both ECE and dieckol possess considerable potential as effective antiviral agents against ZIKV infection.

## Figures and Tables

**Table 1:** The calculated binding energies of dieckol to the three main Zika virus proteins.

Energy (kcal/mol)	Envelope Protein	NS2B/NS3	RdRp
-CDOCKER energy	35.42	52.92	−22.80
-CDOCKER interaction energy	75.44	101.44	41.28
Binding energy ^(1)^	−438.09	−1040.51	−1043.40
Complex energy	−13,786.30	−7676.60	−33,516.60
Protein energy	−13,522.20	−6864.92	−32,645.00
Ligand energy	173.99	228.83	171.75

^(1)^ Energy_Binding_ = Energy_Complex_ – Energy_Protein_ – Energy_Ligand_.

**Table 2:** Primer sequences for qPCR.

Gene	Sequence	Direction
ZIKV NS1	5′-CRA CTA CTG CAA GYG GAA GG-3′	Forward
	5′-GCC TTA TCT CCA TTC CAT ACC-3′	Reverse
TNF-α	5′-CCA ACT GTC ACT CAT TGC TGA-3′	Forward
	5′-CTT TTG GTT ACT TTT CCC CTA TCC-3′	Reverse
IFIT1	5′-GGA TTC TGT ACA ATA CAC TAG AAA CCA-3′	Forward
	5′-CTT TTG GTT ACT TTT CCC CTA TCC-3′	Reverse
IFIT2	5′-ATC CCC CAT CGC TTA TCT CT-3	Forward
	5′-CCACCTCAATTAATCAGGCACT-3′	Reverse

## Data Availability

The original contributions presented in the study are included in the article; further inquiries can be directed to the corresponding author.

## Supplementary Materials

The following supporting information can be downloaded at: https://www.mdpi.com/article/10.3390/ijms252413694/s1.

## References

[1] Pereira R.S., Santos F.C.P., Campana P.R.V., Costa V.V., de Pádua R.M., Souza D.G., Teixeira M.M., Braga F.C. (2023). Natural products and derivatives as potential Zika virus inhibitors: A comprehensive review. Viruses.

[2] Vázquez-Calvo Á., Jiménez de Oya N., Martín-Acebes M.A., Garcia-Moruno E., Saiz J.-C. (2017). Antiviral properties of the natural polyphenols delphinidin and epigallocatechin gallate against the flaviviruses West Nile virus, Zika virus, and dengue virus. Front. Microbiol..

[3] Kang N., Kim E.-A., Park A., Heo S.-Y., Heo J.-H., Heo S.-J. (2024). Antiviral Potential of Fucoxanthin, an Edible Carotenoid Purified from Sargassum siliquastrum, against Zika Virus. Mar. Drugs.

[4] Sadeghieh T., Sargeant J.-M., Greer A.-L., Berke O., Dueymes G., Gachon P., Ogden N.-H., Ng V. (2021). Zika virus outbreak in Brazil under current and future climate. Epidemics.

[5] Reis A.C.C., Silva B.M., de Moura H.M.M., Pereira G.R., Brandão G.C. (2020). Anti-Zika virus activity and chemical characterization by ultra-high performance liquid chromatography (UPLC-DAD-UV-MS) of ethanol extracts in Tecoma species. BMC Complement. Altern. Med..

[6] Acquadro S., Civra A., Cagliero C., Marengo A., Rittà M., Francese R., Sanna C., Bertea C., Sgorbini B., Lembo D. (2020). Punica granatum leaf ethanolic extract and ellagic acid as inhibitors of Zika virus infection. Planata Med..

[7] World Health Organization. https://www.who.int/activities/prioritizing-diseases-for-research-and-development-in-emergency-contexts.

[8] Zhao J.-H., Wang Y.-W., Yang J., Tong Z.-J., Wu J.-Z., Wang Y.-B., Wang Q.-X., Li Q.-Q., Yu Y.-C., Leng X.-J. (2023). Natural products as potential lead compounds to develop new antiviral drugs over the past decade. Eur. J. Me. Chem..

[9] Santos L.H., Rocha R.E., Dias D.L., Ribeiro B.M., Serafim M.S.M., Abrahão J.S., Ferreira R.S. (2023). Evaluating Known Zika Virus NS2B-NS3 Protease Inhibitor Scaffolds via In Silico Screening and Biochemical Assays. Pharmaceuticals.

[10] Feng Y. (2024). Recent advances in the study of zika virus structure, drug targets, and inhibitors. Front. Pharmacol..

[11] Delgado-Maldonado T., Moreno-Herrera A., Pujadas G., Vázquez-Jiménez L.K., González-González A., Rivera G. (2023). Recent advances in the development of methyltransferase (MTase) inhibitors against (re)emerging arboviruses diseases dengue and Zika. Eur. J. Med. Chem..

[12] Byler K.G., Ogungbe I.V., Setzer W.N. (2016). Modelling, In-silico screening for anti-Zika virus phytochemicals. J. Mol. Graph. Medel..

[13] Gonzalez G., MacCann R., Garcia Leon A.A., Carr M., Feeney E., Yousif O., Cotter A., de Barra E., Sadlier C., Doran P. (2023). Dysregulated early transcriptional signatures linked to mast cell and interferon responses are implicated in COVID-19 severity. Front. Immunol..

[14] Oyler-Yaniv J., Oyler-Yaniv A., Maltz E., Wollman R. (2021). TNF controls a speed-accuracy tradeoff in the cell death decision to restrict viral spread. Nat. Commun..

[15] Ruby J., Bluethmann H., Peschon J.J. (1997). Antiviral activity of tumor necrosis factor (TNF) is mediated via p55 and p75 TNF receptors. J. Exp. Med..

[16] Vladimer G.I., Górna M.W., Superti-Furga G. (2014). IFITs: Emerging roles as key anti-viral proteins. Front. Immunol..

[17] Xavier J.A., Santos J.C., Nova M.A.V., Gonçalves C.M., Borbely K.S., Pires K.S., dos Santos F.A.R., Valentim I.B., Barbosa J.H., da Silva F.C. (2022). Anti-Zika virus effects, placenta protection and chemical composition of Passiflora edulis seeds ethanolic extract. J. Braz. Chem. Soc..

[18] Lee J.-H., Kim T.-K., Kang M.-C., Park M.-K., Park S.-H., Choi J.-S., Choi Y.-S. (2024). Effect of Crude Polysaccharides from Ecklonia cava Hydrolysate on Cell Proliferation and Differentiation of Hanwoo Muscle Stem Cells for Cultured Meat Production. Foods.

[19] Kang M.-C., Kim K.-N., Wijesinghe W., Yang X., Ahn G., Jeon Y.-J. (2014). Protective effect of polyphenol extracted from Ecklonia cava against ethanol induced oxidative damage in vitro and in zebrafish model. J. Funct. Foods.

[20] Suryaningtyas I.T., Lee D.-S., Je J.-Y. (2024). Brown Algae Ecklonia cava Extract Modulates Adipogenesis and Browning in 3T3-L1 Preadipocytes through HO-1/Nrf2 Signaling. Mar. Drugs.

[21] Karadeniz F., Kang K.-H., Park J.W., Park S.-J., Kim S.-K. (2014). Anti-HIV-1 activity of phlorotannin derivative 8, 4‴-dieckol from Korean brown alga Ecklonia cava. Biosci. Biotechnol. Biocehm..

[22] Montenegro-Landívar M.F., Tapia-Quirós P., Vecino X., Reig M., Valderrama C., Granados M., Cortina J.L., Saurina J. (2021). Polyphenols and their potential role to fight viral diseases: An overview. Sci. Total Environ..

[23] Kim Y.-S., Kim K.A., Seo H.-Y., Kim S.H., Lee H.M. (2024). Antioxidant and anti-hepatitis A virus activities of Ecklonia cava Kjellman extracts. Heliyon.

[24] Kwon H.-J., Ryu Y.B., Kim Y.-M., Song N., Kim C.Y., Rho M.-C., Jeong J.-H., Cho K.-O., Lee W.S., Park S.-J. (2013). In vitro antiviral activity of phlorotannins isolated from Ecklonia cava against porcine epidemic diarrhea coronavirus infection and hemagglutination. Bioorg. Med. Chem..

[25] Yang H.-K., Jung M.-H., Avunje S., Nikapitiya C., Kang S.Y., Ryu Y.B., Lee W.S., Jung S.-J. (2018). Efficacy of algal Ecklonia cava extract against viral hemorrhagic septicemia virus (VHSV). Fish Shellfish Immunol..

[26] Rajan D.K., Mohan K., Zhang S., Ganesan A.R. (2021). Dieckol: A brown algal phlorotannin with biological potential. Biomed. Pharmacother..

[27] Okechukwu Q.N., Adepoju F.O., Kanwugu O.N., Adadi P., Serrano-Aroca Á., Uversky V.N., Okpala C.O.R. (2024). Marine-Derived Bioactive Metabolites as a Potential Therapeutic Intervention in Managing Viral Diseases: Insights from the SARS-CoV-2 In Silico and Pre-Clinical Studies. Pharmaceuticals.

[28] Aatif M., Muteeb G., Alsultan A., Alshoaibi A., Khelif B.Y. (2021). Dieckol and its derivatives as potential inhibitors of SARS-CoV-2 spike protein (UK strain: VUI 202012/01): A computational study. Mar. Drugs.

[29] Park J.-Y., Kim J.H., Kwon J.M., Kwon H.-J., Jeong H.J., Kim Y.M., Kim D., Lee W.S., Ryu Y.B. (2013). Dieckol, a SARS-CoV 3CL(pro) inhibitor, isolated from the edible brown algae Ecklonia cava. Bioorg. Med. Chem..

[30] Zhu Y., Liang M., Yu J., Zhang B., Zhu G., Huang Y., He Z., Yuan J. (2023). Repurposing of doramectin as a new anti-zika virus agent. Viruses.

[31] Upadhyay A.K., Cyr M., Longenecker K., Tripathi R., Sun C., Kempf D.J. (2017). Crystal structure of full-length Zika virus NS5 protein reveals a conformation similar to Japanese encephalitis virus NS5. Acta Crystallogr. F Struct. Biol. Commun..

[32] Dai L., Song J., Lu X., Deng Y.-Q., Musyoki A.M., Cheng H., Zhang Y., Yuan Y., Song H., Haywood J. (2016). Structures of the Zika virus envelope protein and its complex with a flavivirus broadly protective antibody. Cell Host Microbe..

[33] Cirne-Santos C., de Souza Barros C., Nogueira C.C.R., Campos R.M., Teixeira V., Ferreira D., de Palmer Paixão I.C.N. (2017). Inhibition of Zika virus by marine algae. Preprints.

[34] Cirne-Santos C.C., Barros C.D.S., Gomes M.W., Gomes R., Cavalcanti D.N., Obando J.M., Ramos C.J., Villaça R.C., Teixeira V.L., de P Paixão I.C.N. (2021). In vitro antiviral activity against zika virus from a natural product of the Brazilian brown seaweed Dictyota menstrualis. Acta Virol..

[35] Saifulazmi N.F., Rohani E.R., Harun S., Bunawan H., Hamezah H.S., Muhammad N.A.N., Azizan K.A., Ahmed Q.U., Fakurazi S., Mediani A. (2022). A Review with Updated Perspectives on the Antiviral Potentials of Traditional Medici-nal Plants and Their Prospects in Antiviral Therapy. Life.

[36] Kim D.-Y., Park H.-J., Yun C.-I., Kim Y.-J. (2024). Method development and validation of phloroglucinol and dieckol in Ecklonia cava using HPLC–DAD. Food Sci. Biotechnol..

[37] Fong Y.D., Chu J.J.H. (2022). Natural products as zika antivirals. Med. Res. Rev..

[38] Fink S.L., Vojtech L., Wagoner J., Slivinski N.S., Jackson K.J., Wang R., Khadka S., Luthra P., Basler C.F., Polyak S.J. (2018). The antiviral drug arbidol inhibits Zika virus. Sci. Rep..

[39] García-Lozano M.d.R., Dragoni F., Gallego P., Mazzotaa S., López-Gómez A., Boccuto A., Martínez-Cortés C., Rodríguez-Martínez A., Pérez-Sánchez H., Campo J.A.D. (2023). Piperazine-derived small molecules as potential Flaviviridae NS3 protease inhibitors. In vitro antiviral activity evaluation against Zika and Dengue viruses. Bioorg. Chem..

[40] Heo S.-J., Ko S.-C., Cha S.-H., Kang D.-H., Park H.-S., Choi Y.-U., Kim D., Jung W.-K., Jeon Y.-J. (2009). Effect of phlorotannins isolated from Ecklonia cava on melanogenesis and their protective effect against photo-oxidative stress induced by UV-B radiation. Vitr. Toxicol.

[41] Kim E.-A., Han E.-J., Kim J., Fernando I.P.S., Oh J.-Y., Kim K.-N., Ahn G., Heo S.-J. (2022). Anti-allergic effect of 3,4-dihydroxybenzaldehyde isolated from Polysiphonia morrowii in IgE/BSA-stimulated mast cells and a passive cutaneous anaphylaxis mouse model. Mar. Drugs.

[42] Kim E.-A., Kang N., Heo S.-Y., Oh J.-Y., Lee S.-H., Cha S.-H., Kim W.-K., Heo S.-J. (2023). Antioxidant, antiviral, and anti-inflammatory activities of lutein-enriched extract of Tetraselmis species. Mar. Drugs.

