# Supplementary material for: Antiviral Activity of Ecklonia cava Extracts and Dieckol Against Zika Virus
Source: Int J Mol Sci. 2024 Dec 21;25(24):13694. doi: 10.3390/ijms252413694 (PMC11728237; doi:10.3390/ijms252413694)
Supplement: Supplementary file 1 [file ijms-25-13694-s001.zip › ijms-3349533-supplementary.pdf]

Supplementary materials

Anti-viral activity of *Ecklonia cava* extracts and dieckol against Zika virus

Eun-A Kim[1†], Nalae Kang[1†], Jun-Ho Heo[1], Areumi Park[1], Seong-Yeong Heo[1], Hyun-Soo Kim[2], Soo-Jin Heo[1,3*]

1 Jeju Bio Research Center, Korea Institute of Ocean Science and Technology (KIOST), Jeju 63349, Republic of Korea; euna0718@kiost.ac.kr (E.-A.K.); nalae1207@kiost.ac.kr (N.K.); unknown0713@kiost.ac.kr (J.-H.H.); areumi1001@kiost.ac.kr (A.P.); syheo@kiost.ac.kr (S.-Y.H.)

2 Department of Seafood Science and Technology, The Institute of Marine Industry, Gyeongsang National University, Tongyeong 53064, Republic of Korea; gustn783@gnu.ac.kr

3 Department of Marine Biology, University of Science and Technology, Daejeon 34113, Republic of Korea

* Correspondence: sjheo@kiost.ac.kr

† These authors contributed equally to this work.

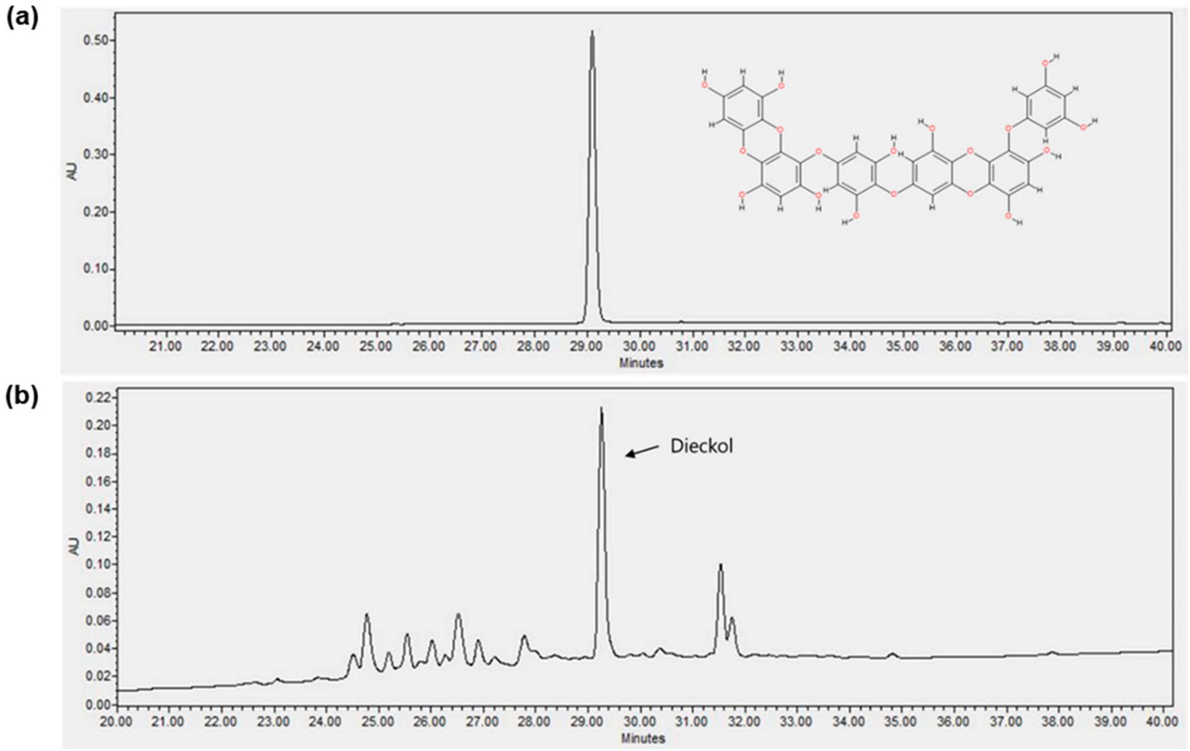

Figure S1. HPLC chromatograms of (a) dieckol standard and (b) *Ecklonia cava* ethanol extract (ECE).
